# Identification of Potentially Inappropriate Medications for Adults Below 65 Years: Protocol for a Modified Delphi Study

**DOI:** 10.2196/92082

**Published:** 2026-07-03

**Authors:** Eva Filosa, Nicole Schönenberger, Utibe R Essien, Camille Gagnon, Danijela Gnjidic, Lisa Kouladjian O’Donnell, Elizabeth Manias, Lisa M McCarthy, Frank Moriarty, Jérôme Nguyen-Soenen, Sion Scott, Michael A Steinman, Petra A Thürmann, Marnie Goodwin Wilson, Bianca J Gutman, Arielle Mendel, Emilie Trinh, Todd C Lee, Emily G McDonald

**Affiliations:** 1 Division of Clinical and Translational Research, Faculty of Medicine and Health Sciences, McGill University, 1001 Decarie Boulevard, Montreal, QC, H4A 3J1, Canada, 1 (514) 934-1934 ext 36134; 2 Clinical Pharmacology and Toxicology, Department of General Internal Medicine, University Hospital of Bern, Bern, Switzerland; 3 Graduate School for Health Sciences, University of Bern, Bern, Switzerland; 4 Division of General Internal Medicine & Health Services Research, David Geffen School of Medicine, University of California, Los Angeles, Los Angeles, CA, United States; 5 Canadian Medication Appropriateness and Deprescribing Network, Centre de Recherche, Institut Universitaire de Gériatrie de Montréal, Montreal, QC, Canada; 6 School of Pharmacy, Faculty of Medicine and Health, The University of Sydney, Camperdown, New South Wales, Australia; 7 Faculty of Medicine, Nursing and Health Sciences, Monash University, Clayton, Victoria, Australia; 8 Leslie Dan Faculty of Pharmacy, University of Toronto, Toronto, ON, Canada; 9 School of Pharmacy and Biomolecular Sciences, Royal College of Surgeons in Ireland, Dublin, Ireland; 10 Department of General Practice, Faculty of Medicine, Nantes Université, Nantes, France; 11 POPS, SFR ICAT, Université d’Angers, Angers, France; 12 School of Health Sciences, University of East Anglia, Norwich, United Kingdom; 13 Division of Geriatrics, University of California, San Francisco, San Francisco, CA, United States; 14 Department of Clinical Pharmacology, Faculty of Health, Witten/Herdecke University, Witten, Germany; 15 Division of General Internal Medicine and Critical Care, University of British Columbia, Vancouver, BC, Canada; 16 Patient Partner, Montreal, QC, Canada; 17 Division of Rheumatology, Department of Medicine, McGill University Health Centre, Montreal, QC, Canada; 18 Division of Nephrology, Department of Medicine, McGill University Health Centre, Montreal, QC, Canada; 19 Division of Infectious Diseases, Department of Medicine, McGill University Health Centre, Montreal, QC, Canada; 20 Clinical Practice Assessment Unit, Division of Internal Medicine, McGill University, Montreal, QC, Canada; 21 Division of General Internal Medicine, Department of Medicine, McGill University Health Centre, Montreal, QC, Canada

**Keywords:** Delphi technique, consensus development, study protocol, deprescribing, potentially inappropriate medications, polypharmacy, medication appropriateness, adult population

## Abstract

**Background:**

Polypharmacy is increasingly common across all age groups and is often associated with the use of potentially inappropriate medications (PIMs), where harms may outweigh benefits, contributing to increased adverse drug events, reduced quality of life, and rising health care costs. However, existing deprescribing guidelines and PIM criteria, such as the Beers Criteria and STOPP/START (Screening Tool of Older Persons’ Prescriptions/Screening Tool to Alert to Right Treatment), primarily target older adults, overlooking the risks faced by younger populations.

**Objective:**

This study aims to develop an international consensus-based list of PIMs for adults aged 18 to 65 years through a modified Delphi process, addressing a critical gap in medication safety and deprescribing guidance.

**Methods:**

A 14-member international steering committee developed a preliminary list of candidate PIMs through a literature review and expert input. An international, interdisciplinary Delphi panel of 30 to 40 participants will evaluate this list across 3 survey rounds. Panelists will rate the relevance of potential PIMs using predefined criteria, including the balance of benefits and harms, patient preferences, and the availability of alternatives. Consensus will be defined as a median rating of ≥4 (for inclusion) or ≤2 (for exclusion) on a 5-point Likert scale (1=not relevant to 5=extremely relevant), with an IQR width of ≤1 and at least 70% directional agreement. Items not meeting thresholds will be carried forward to subsequent rounds for reconsideration. Quantitative analyses will summarize ratings and agreement levels, while qualitative free-text responses will undergo content analysis to provide context and capture nuanced perspectives. The process will yield a prioritized list of PIMs for adults aged 18 to 65 years.

**Results:**

Panel recruitment was completed between September and October 2025, with 36 participants. Three rounds of data collection were completed between October 2025 and January 2026. Interim analyses were conducted to inform structured feedback between rounds. Final data analysis is ongoing, and consensus results are expected to be reported in late 2026.

**Conclusions:**

This Delphi process will yield a consensus-based list of PIMs for adults aged 18 to 65 years, informing the development of an international guideline to support safer prescribing practices and to expand deprescribing efforts beyond geriatric care.

## Introduction

### Overview

Polypharmacy, often defined as the concurrent use of 5 or more medications, is a growing concern in health care, affecting a significant proportion of adults [1]. The more medications a patient takes, the greater the likelihood that one or more may be potentially inappropriate medications (PIMs)—medications for which the potential harms outweigh the benefits [2]. The use of PIMs contributes to adverse drug events, reduced quality of life, and increased health care costs [3], highlighting an essential need for strategies to optimize appropriate medication use [4].

Despite rising rates of polypharmacy and adverse drug events among all age groups [5-7], existing PIM guidelines predominantly address older adults (aged ≥65 years) [8-10]. Guidelines such as the American Geriatrics Society Beers Criteria were initially developed for this population, as they are more likely to experience polypharmacy and drug-related harms [8]. For example, one in 4 community-dwelling older adults in Canada takes 10 or more different classes of medications [11], which is associated with adverse drug reactions, impaired balance and cognition, and increased hospitalizations [12]. However, the accumulation of medications leading to polypharmacy often begins earlier in life due to the increasing prevalence of chronic disease and the clinical management of patients with multiple comorbidities [1]. As a result, adults of all ages may face unnecessary risks from PIMs [13,14].

Existing PIM criteria for older adults cannot be generalized to younger populations. Many medications are considered inappropriate only under specific conditions—such as prolonged use, the presence of certain comorbidities, or interactions with other drugs—or become problematic primarily due to age-related physiological changes or cognitive vulnerability [13]. Conversely, some medications may pose inappropriate risks for adults when considering individual and clinical circumstances, regardless of age. For example, the prolonged use of proton pump inhibitors may increase the risk of bone fractures and nutrient deficiencies in adults of any age, particularly when used without clear indication or beyond recommended durations [15,16]. A consensus list adapted to the clinical realities and risk profiles of younger and middle-aged adults is therefore needed to ensure relevance and utility in practice. Addressing this gap could proactively improve medication safety by preventing PIM exposure earlier in life and enabling deprescribing before adverse events occur.

This study employs a modified Delphi method—adapted to better reflect the clinical complexity of medication appropriateness decisions—a structured, iterative process used to achieve consensus among a panel of experts [17,18]. This approach is particularly effective for addressing complex or emerging issues where evidence is limited. In a Delphi process, experts participate in multiple rounds of confidential surveys, providing input, reviewing group feedback, and refining their opinions until consensus is reached [17-20]. By engaging a diverse panel of international experts, this project aims to develop a consensus-based list of PIMs relevant to adults aged 18 to 65 years, thereby extending the scope of deprescribing efforts beyond geriatric populations and promoting safer and more appropriate medication use across adulthood.

### Objective

The primary objective of this study is to develop a consensus-based list of PIMs for adults aged 18 to 65 years using a modified Delphi process. This list will inform the creation of a clinical guideline for this population. By focusing on common medications with the highest potential for harm or lack of utility, this study seeks to ensure that younger patients benefit from proactive deprescribing strategies, complementing existing recommendations for older adults and addressing a critical gap in the management of polypharmacy.

## Methods

### Contributors

#### Steering Committee

A steering committee comprising 14 individuals was formed to define the research objectives, oversee the study design, guide the modified Delphi process, and develop a preliminary list of PIMs to be evaluated. This international, multidisciplinary team was intentionally selected to ensure demographic, geographic, and professional diversity. Members included health care professionals (eg, general practitioners, specialist physicians, pharmacists, and nurses), researchers, and individuals with lived experience. The steering committee included a patient partner to ensure that patient perspectives informed the study design and the development of the preliminary PIMs list. Selection criteria considered gender, age, profession, years of experience, and geographic location.

#### Modified Delphi Panel

The modified Delphi panel will aim to include approximately 30 to 40 experts representing key stakeholders involved in the development and implementation of medication guidelines [17,21]. This number was selected to ensure diverse representation while remaining consistent with prior evidence, showing that reliable outcomes can be achieved with smaller panels (eg, approximately 20‐25 experts) when inclusion criteria are rigorous [22]. The slightly larger target also accounts for potential attrition across Delphi rounds. Panelists will be selected based on their expertise in polypharmacy, PIMs, and deprescribing, as well as prior experience with guideline development. Fields represented will include primary care, internal medicine, geriatrics, clinical pharmacology, pharmacy, and related disciplines. Additionally, efforts will be made to include perspectives that may not align with the steering committee’s initial views to ensure the representation of diverse opinions [17].

#### Recruitment

Both the steering committee and modified Delphi panel members will be identified through preexisting deprescribing networks [23-26], personal contacts, and responses to a Canadian Medication Appropriateness and Deprescribing Network newsletter call for participation [27]. All communication and recruitment will occur via email, with recruitment emails outlining the study’s purpose, process, and expectations.

### Development of the Initial PIM List

The initial list of PIMs was developed through a focused literature search and narrative synthesis, drawing on existing deprescribing guidelines, national and international PIM criteria (eg, Beers Criteria, STOPP/START [Screening Tool of Older Persons’ Prescriptions/Screening Tool to Alert to Right Treatment], FORTA [Fit for the Aged], Prescrire, etc) [8-10,28,29], and peer-reviewed publications focusing on medication safety in adults aged 18 to 65 years. To ensure clinical relevance and completeness, several experts with different professional backgrounds contributed sequentially to the review and refinement of the draft list, including steering committee members, 2 licensed pharmacists, and a hospital-based general internist. This process involved the addition of medications or classes not previously captured, clarification of subcategories (eg, formulation or indication), and identification of scenarios where appropriateness may vary depending on the clinical context.

The resulting list (see Multimedia Appendix 1) was then pilot-tested among 3 individuals (EF, NS, and EGM) and further adapted based on feedback from this pilot phase to ensure usability and clarity for the Delphi panel. It forms the basis for the round 1 questionnaire in the Delphi process.

### Data Collection

#### Survey/Questionnaire Administration

The modified Delphi process will be conducted using REDCap (Research Electronic Data Capture), hosted at McGill University [30]. Surveys will be administered in a series of 3 predetermined rounds, with each survey remaining open for approximately 2 weeks. During this period, participants will receive email reminders to encourage timely responses and reduce attrition rates between rounds. The process is expected to last a total of 3 months.

Following each survey round, the research team will analyze the results to assess levels of consensus. Summary findings will be compiled and shared with participants in subsequent rounds as part of the iterative feedback process, allowing panelists to reflect on and refine their responses in pursuit of a well-informed consensus.

#### Round 1

Participants will be introduced to the study’s purpose, objectives, and process via an introductory letter, which will outline the criteria used for evaluating medications. For instance, participants will be asked to evaluate the medications using four criteria: (1) importance to guideline inclusion, (2) balance of benefits and harms, (3) relevance to patient preferences and values, and (4) availability of alternatives.

Participants will complete a questionnaire in this round containing different sections. The first section will include demographics, including age, geographic location, primary role, and years of experience. The second section is a medication questionnaire designed to assess the relevance of medications or medication classes for inclusion in a list of PIMs for adults aged 18 to 65 years.

Two types of rating formats will be used, depending on the nature of the medication or medication class. This approach contextually reflects the complexity of medication use in practice and the variety of medications and classes being addressed. For some medications, panelists will be asked to evaluate relevance across multiple clinical contexts, including general use (often inappropriate in most cases), use in combination with another medication, use in a specific medical condition, or use over the long term. For each of these contexts, panelists will rate the relevance of the medication or medication class as a PIM using a 5-point Likert scale: “1—not relevant,” “2—slightly relevant,” “3—moderately relevant,” “4—very relevant,” and “5—extremely relevant,” with optional free-text boxes for explanations or suggestions of additional medications to be considered in subsequent rounds. Panelists will also have the option to select “Insufficient knowledge to evaluate” if they do not feel comfortable assessing a particular medication or context. For other medications or medication classes, a specific clinical context may already be provided, and panelists will be asked to assess the overall relevance of the medication as a PIM within that context using the same 5-point Likert scale. An open-ended comment box will accompany each item, allowing panelists to optionally provide explanations, examples, or additional considerations to support their ratings.

The output of round 1 will include a set of medications and medication classes for which consensus was achieved and retained for inclusion, newly suggested medications to be introduced and rated for the first time in round 2, and a subset of medications with a lack of consensus that will be carried forward into round 2 for further discussion. In addition, context-specific concerns raised by panelists may be integrated into subsequent rounds for further evaluation (for additional information, refer to Multimedia Appendix 2).

#### Round 2

In round 2, participants will be provided with personalized feedback from round 1, including visual summaries of the group’s responses (eg, histograms or forest plots of median Likert scores), and a summary of which items achieved consensus for inclusion in round 1. Participants will be asked to reassess a revised list of medications and medication classes, with and without context-specific scenarios, using the same 5-point Likert scale. This revised list will include items that did not reach consensus in round 1 and are being reevaluated with the aim of achieving agreement among panelists. Participants will have the opportunity to explain any changes in their ratings and to share additional comments. The collected data will be analyzed to identify key themes and further refine the prioritization of medications. Additional details can be found in Multimedia Appendix 2.

#### Round 3

In round 3, participants will once again receive feedback summarizing the results from the previous round. They will be asked to reassess a further refined list of medications and medication classes, with and without context-specific scenarios, that have not yet achieved consensus. Using the same 5-point Likert scale as in previous rounds, participants will be invited to reconsider their ratings in light of group trends and to provide explanations for any changes, along with any additional comments. This final round aims to promote convergence of expert opinion and establish consensus on the relevance of each medication or class as a PIM for adults aged 18 to 65 years. Additional details are provided in Multimedia Appendix 2.

### Data Analysis

#### Demographics

The collected demographic information will be summarized using descriptive statistics (eg, frequencies, percentages, and where applicable, means and SDs).

#### Quantitative Analysis

The analysis for this study will be conducted across the 3 rounds of the modified Delphi process, with each round building on the previous results to achieve a consensus on a list of PIMs for adults aged 18 to 65 years. Statistical analyses will be performed using Microsoft Excel.

In this study, consensus is defined as the combination of directional agreement, indicated by the median rating, and clustering of responses, indicated by an IQR width of 1 or less, along with a predefined proportion of similar ratings. Consensus for inclusion requires high ratings (median ≥4), and consensus for exclusion requires low ratings (median ≤2), each combined with tight clustering and at least 70% of participants rating ≥4 or ≤2, respectively. Disagreement is indicated by a wide dispersion of ratings (IQR>1), reflecting variability in panelists’ views. Items for which consensus is not achieved are considered to have “no consensus” and will be handled according to the criteria outlined below [31,32].

In round 1, ratings will be summarized using the median and IQR values. A consensus will be determined as above. Items meeting consensus for inclusion will be retained, whereas those meeting consensus for exclusion will be removed from future consideration. Items that do not meet the predefined criteria for either inclusion or exclusion, including those with a median rating of 3 or a wider dispersion (IQR >1), will be carried forward to the next round for reevaluation. Responses marked as “Insufficient knowledge to evaluate” will be excluded from consensus calculations but reported separately. If over 25% of participants indicate insufficient knowledge for a particular item, it will be flagged and considered by the steering committee for potential exclusion or modification in the next round. New medications suggested by participants will be included in round 2 if the same medication is independently proposed by at least 2 panelists.

In round 2, medications and medication classes that did not reach consensus in round 1 will be reassessed. The same quantitative approach will be applied to determine consensus. Medications that still demonstrate disagreement will be retained for a final round of evaluation. Newly suggested items introduced through free-text responses in round 1 will be rated for the first time in round 2 and analyzed using the same criteria.

In round 3, participants will again reassess items that have not yet reached consensus. To assess overall group agreement and convergence across participants in this final round, the Kendall coefficient of concordance (W) may also be calculated, with a value of 0.7 or higher indicating strong agreement among panelists [18,31]. Items with a final median rating of 3, a wide dispersion of responses (IQR >1), or failure to meet the threshold for either inclusion or exclusion will be classified as “no consensus” and excluded from the final prioritized list. These items will be documented separately and may be subject to additional discussion and evaluation by the steering committee, if necessary.

#### Qualitative Analysis

Qualitative free-text responses collected across all 3 rounds will be analyzed using the conventional content analysis, an inductive approach in which codes and themes emerge directly from the data rather than being imposed a priori [33,34]. Two independent coders will categorize comments to identify recurring themes and patterns, with discrepancies resolved through discussion. Qualitative themes will be considered alongside quantitative consensus results to provide a more complete interpretation of panelists’ perspectives, particularly for items with divergent expert opinions or those near consensus thresholds.

### Addressing Attrition

To minimize participant attrition, participants were informed of the study timeline and expected commitment prior to enrollment. Surveys were designed with several features to reduce response burden: sections were divided to allow progress to be saved and resumed, items were grouped by therapeutic area, an estimated completion time was provided at the outset, and the survey was optimized for mobile access. Panelists also had the option to select “Insufficient knowledge to evaluate” for any item they did not feel comfortable assessing. Two reminder emails were sent during each round to encourage timely and continued participation.

To preserve the integrity of the Delphi process, only participants who complete each round will be allowed to proceed to subsequent rounds. No data imputation will be performed for participants who drop out in later rounds to avoid introducing bias or uncertainty from imputed values. This approach also simplifies analysis, reducing the risk of misinterpretation [18,31].

### Ethical Considerations

Ethics approval has been obtained from the Research Ethics Board of the Research Institute of the McGill University Health Centre in Montréal, Québec (approval number 2025‐11172). Prior to participation, all panelists will be provided with an informed consent form outlining the study’s purpose, procedures, risks, benefits, and confidentiality measures. Participants will be required to review and sign the informed consent form before receiving access to the first survey round.

All data collected during the study will be stored and managed using REDCap hosted at McGill University [30]. Identifying information (eg, names and email addresses) will be stored separately from survey response data and will only be accessible to authorized members of the research team. This information will be used exclusively for administrative purposes, such as sending reminders and sharing round-specific feedback.

Data will be coded prior to analysis and dissemination. The Delphi process will remain anonymous among participants; panelists will not be informed of the identities or individual responses of others. Feedback between rounds will be presented in aggregate form using deidentified data (eg, medians and IQRs). At no point will individual responses be linked to identifying information in any communications with participants or in any published reports or presentations.

All study data will be retained for a period of 7 years, following study completion, in accordance with institutional guidelines. Only authorized members of the study team will have access to the study key and identifiable data.

This study was reported in accordance with the ACCORD (Accurate Consensus Reporting Document) guideline for health care–based consensus studies [20]. An ACCORD guideline checklist for reporting consensus methods is provided in Checklist 1, which aided in the reporting of this study protocol.

### Proposed Timeline

**Figure 1.**
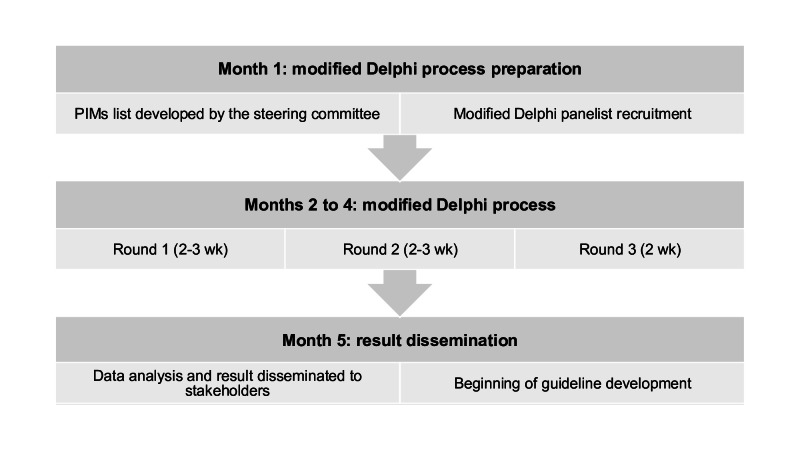
Timeline of the modified Delphi process for the potentially inappropriate medication (PIM) list development.

The tentative timeline for this Delphi study spans 5 months (Figure 1).

Month 1: The initial list of PIMs will be generated, and recruitment for the Delphi panel will be conducted.

Months 2 to 4:

- Round 1: The first round of the modified Delphi process will be conducted. Data will be analyzed, and the priority list will be revised. This round is expected to take 2 to 3 weeks.
- Round 2: The second round of the modified Delphi process will be conducted. Data will be analyzed, and the priority list will be refined. This round will also take 2 to 3 weeks.
- Round 3: The final round will focus on achieving consensus on the top priority PIMs for guideline development. This round will take approximately 2 weeks.

Month 5: Comprehensive data analysis will be completed, and the results will be compiled into a final report. The findings will be disseminated to stakeholders and will support subsequent guideline development.

This timeline ensures a structured and efficient progression through each phase of the Delphi process while allowing sufficient time for analysis and feedback integration. The exact timeline outlined for this study is subject to change based on participant availability, unforeseen delays, and the iterative nature of the Delphi process.

## Results

Panel recruitment was completed between September and October 2025, with 36 participants. Three rounds of data collection were completed between October 2025 and January 2026. Interim analyses were conducted to inform structured feedback between rounds. Final data analysis is ongoing, and consensus results are expected to be reported in Fall 2026.

## Discussion

### Anticipated Findings

This study is anticipated to generate a consensus-based list of PIMs for adults aged 18 to 65 years, addressing a critical gap in polypharmacy research. While existing PIM criteria have historically focused on older adults [8-10,29], no prior effort has systematically identified inappropriate medications for younger populations, despite the common use of many potentially problematic medications in this age group [13]. By identifying medications that warrant reassessment in adults below 65 years, this research promotes a more comprehensive, proactive, and inclusive approach to safer medication use and deprescribing across the adult lifespan.

The resulting list is intended for use by clinicians, including physicians, nurse practitioners, and pharmacists, who may not have formal training in deprescribing outside of geriatric care. It aims to serve as a practical reference to help identify candidate medications for review or discontinuation in adult patients. The 4 evaluation criteria considered throughout the Delphi process (ie, importance to guideline inclusion, balance of benefits and harms, relevance to patient preferences and values, and availability of alternatives) will provide structured context for each medication’s inclusion in the final list, with more detailed evidence-based rationale to be developed during the subsequent guideline development phase.

The findings will serve as a foundation for developing an evidence-based clinical guideline, following established principles for guideline development, to support health care providers in assessing and deprescribing PIMs in younger and middle-aged adults [35-40]. The guideline development is anticipated to follow the WikiGuidelines methodology, a collaborative, iterative, and transparent approach that integrates expert consensus, current evidence, and stakeholder input to ensure clinical utility and adaptability over time [40]. For each prioritized PIM, a dedicated section will provide concise, evidence-informed recommendations, presented in a modular format to facilitate integration into diverse clinical settings.

In addition to the overarching guideline, individual deprescribing documents may be developed to further support implementation, tailored to specific clinical contexts or care providers. Ultimately, this work aims to enhance medication safety and improve health outcomes by enabling clinicians and patients to identify PIMs across the lifespan, thereby reducing the risks associated with polypharmacy, including adverse drug reactions, medication burden, and diminished quality of life among adults of all ages.

### Limitations

Several methodological considerations should be acknowledged. First, recruitment through established deprescribing networks may introduce self-selection bias, as participants may already be predisposed toward deprescribing. Second, although study materials were available in both English and French, the process was limited to these 2 languages, which may not fully capture the scope of perspectives across the international panel. Third, while consensus thresholds used in this study are consistent with published Delphi methodology, no universally accepted statistical cutoffs exist for Delphi consensus, and the chosen criteria involve an inherent degree of judgment. The detailed interpretation of findings and their limitations will be addressed in the forthcoming paper.

## Supplementary material

10.2196/92082 Multimedia Appendix 1 Preliminary list of potentially inappropriate medications.

10.2196/92082 Multimedia Appendix 2 Sample Delphi survey instruments for rounds 1 to 3.

10.2196/92082 Checklist 1 ACCORD checklist.

## Abbreviations

ACCORD: Accurate Consensus Reporting Document
FORTA: Fit for the Aged
PIM: potentially inappropriate medication
REDCap: Research Electronic Data Capture
STOPP/START: Screening Tool of Older Persons' Prescriptions/Screening Tool to Alert to Right Treatment
