# Supplementary material for: Identification of Potentially Inappropriate Medications for Adults Below 65 Years: Protocol for a Modified Delphi Study
Source: JMIR Res Protoc. 2026 Jul 3;15:e92082. doi: 10.2196/92082 (PMC13331326; doi:10.2196/92082)
Supplement: Multimedia Appendix 1 [file resprot-v15-e92082-s001.docx]

| **Medication/Medication Class** | **Category** |
| --- | --- |
| **Amantadine** | CNS |
| The following **antiepileptics**:  - Carbamazepine - Oxcarbazepine | CNS |
| **Antihistamines** (first generation), such as:   - Chlorpheniramine - Diphenhydramine - Doxylamine - Hydroxyzine - Oxomemazine - Promethazine | CNS |
| **Antiparkinsonian agents** with strong anticholinergic activity, such as:  - Benztropine - Trihexyphenidyl | CNS |
| **Antipsychotics** (first generation) for insomnia or off-label uses, such as:   - Chlorpromazine - Haloperidol - Levomepromazine - Zuclopenthixol | CNS |
| **Antipsychotics** (second generation) for insomnia or off-label uses, such as:   - Aripiprazole - Clozapine - Olanzapine - Quetiapine - Risperidone | CNS |
| **Barbiturates**, such as:   - Butalbital - Phenobarbital | CNS |
| **Benzodiazepines** for insomnia, anxiety, post-traumatic stress disorder (PTSD) or panic disorder, such as:  - Clonazepam - Diazepam - Lorazepam - Oxazepam - Temazepam | CNS |
| The following **cognitive enhancers** for early dementia or cognitive impairment:  - Donepezil - Galantamine - Memantine - Rivastigmine | CNS |
| The following **dopamine agonists**:   - Bromocriptine - Cabergoline - Pramipexole | CNS |
| **Gabapentinoids**, such as:   - Gabapentin - Pregabalin | CNS |
| **Monoamine oxidase A (MAO-A) inhibitors**, such as:  - Moclobemide - Toloxatone | CNS |
| **Monoamine oxidase B (MAO-B) inhibitors**, such as:   - Rasagiline - Safinamide - Selegiline | CNS |
| **Phenytoin** | CNS |
| The following **serotonin and noradrenaline (norepinephrine) reuptake inhibitors (SNRIs)**:   - Desvenlafaxine - Duloxetine - Venlafaxine | CNS |
| The following **selective serotonin reuptake inhibitors** **(SSRIs)**:  - Citalopram - Escitalopram | CNS |
| **St. John's Wort (*Hypericum perforatum*)** | CNS |
| **Trazodone** | CNS |
| **Tricyclic antidepressants (TCAs)**, such as:   - Amitriptyline - Amoxapine - Clomipramine - Desipramine - Doxepin - Imipramine - Nortriptyline | CNS |
| **Valproate** | CNS |
| **Z-drugs** **(non-benzodiazepine sedatives/hypnotics)** for insomnia, such as:   - Eszopiclone - Zolpidem - Zopiclone | CNS |
| Low dose **acetyl-salicylic acid (ASA)** for primary prevention | Cardiovascular |
| The following **antianginal agents**:  - Ranolazine - Trimetazidine | Cardiovascular |
| The following **antiarrhythmics** (class I and III):   - Amiodarone - Disopyramide - Dronedarone - Ivabradine | Cardiovascular |
| **Beta-blockers** for hypertension or after myocardial infarction (MI) for greater than 3 years such as:   - Atenolol - Propranolol - Sotalol | Cardiovascular |
| **Central antihypertensives**, such as:   - Clonidine - Guanfacine - Methyldopa | Cardiovascular |
| **Digoxin** | Cardiovascular |
| **Fibrates**, such as:   - Bezafibrate - Ciprofibrate - Fenofibrate - Gemfibrozil | Cardiovascular |
| **Long-acting nitrates**, such as:   - Isosorbide dinitrate - Isosorbide mononitrate - Nitroglycerin patches - Pentaerythritol tetranitrate | Cardiovascular |
| **Non-selective alpha-1 blockers**, such as:   - Doxazosin - Prazosin - Terazosin | Cardiovascular |
| **Olmesartan** | Cardiovascular |
| **Vitamin K antagonists**, except when used for mechanical heart valves, antiphospholipid antibody syndrome (APS), left ventricular thrombus, or in patients with very severe chronic kidney disease, such as:   - Acenocoumarol - Phenprocoumon - Warfarin | Cardiovascular |
| **Acarbose** | Endocrine |
| **Androgens**, such as:  - Methyltestosterone - Testosterone | Endocrine |
| **Calcium supplements** in the absence of specific indications (e.g., osteoporosis, malabsorption, corticosteroid therapy, chronic kidney disease with mineral-bone disorder, documented hypocalcemia) | Endocrine |
| The following systemic **estrogen therapies** (with or without progestin), such as:  - Oral estrogen (e.g., estradiol, conjugated estrogens) - Transdermal estrogen patches | Endocrine |
| **Glinides**, such as:  - Nateglinide - Repaglinide | Endocrine |
| **Gliptins** **(DPP-4 inhibitors)**, such as:   - Alogliptin - Linagliptin - Saxagliptin - Sitagliptin - Vildagliptin | Endocrine |
| **Growth hormones**, such as:  - Mecasermin - Somatropin | Endocrine |
| Use of sliding scale **insulin** without concurrent basal insulin | Endocrine |
| **Iron supplements** if:   - Doses higher than 325 mg/day - Ferric (Fe3+) iron salts (e.g. ferric citrate, ferric sulfate) | Endocrine |
| **Levothyroxine** in subclinical hypothyroidism | Endocrine |
| **Megestrol** | Endocrine |
| **Sulfonylureas**, such as:   - Glibenclamide/glyburide - Gliclazide - Glimepiride | Endocrine |
| **Thiazolidinediones (glitazones)**, such as:   - Pioglitazone - Rosiglitazone | Endocrine |
| **Vitamin D** supplementation in the absence of specific indications (rickets, osteomalacia, osteoporosis, advanced chronic kidney disease ± hyperparathyroidism, hepatic insufficiency, malabsorption, treatment with corticosteroids or bisphosphonates, pregnancy or breastfeeding) | Endocrine |
| **Antacids**, such as:   - Combinations and complexes of aluminium-, calcium-, and magnesium-containing compounds - Combinations of aluminium, calcium, and magnesium salts | Gastrointestinal |
| **Gastrointestinal antispasmodics** with strong anticholinergic effects, such as:  - Atropin - Dicyclomine - Hyoscyamine - Scopolamine - Clidinium | Gastrointestinal |
| **Docusate** | Gastrointestinal |
| **Domperidone** | Gastrointestinal |
| The following **dopamine receptor antagonists**:   - Metoclopramide - Prochlorperazine | Gastrointestinal |
| **Lincalotide** | Gastrointestinal |
| **Loperamide,** use >2 days | Gastrointestinal |
| **Proton pump inhibitors**, long-term use (>8 weeks) in the absence of a valid indication (e.g., erosive esophagitis, Barrett’s esophagus, chronic NSAID or corticosteroid use with GI risk, pathologic hypersecretory conditions), such as:   - Dexlansoprazole - Esomeprazole - Lansoprazole - Omeprazole - Pantoprazole - Rabeprazole | Gastrointestinal |
| **Prucalopride** | Gastrointestinal |
| **Stimulant laxatives**, such as:  - Bisacodyl - Senna - Sodium picosulfate | Gastrointestinal |
| **Alpha-1 blockers**, such as:  - Alfuzosin - Doxazosin - Tamsulosin - Terazosin | Genitourinary |
| **Mirabegron** | Genitourinary |
| **Nitrofurantoin** when used long-term | Genitourinary |
| **Oral antimuscarinics**, such as:  - Oxybutynin - Solifenacin - Tolterodine - Trospium | Genitourinary |
| **Vasopressin analogues** for incontinence/increased urinary frequency, such as:   - Desmopressin - Terlipressin | Genitourinary |
| **Antitussives** (opioid-based and non-opioid), such as:  - Butamirate - Codeine - Dextromethorphan | Respiratory |
| **Inhaled corticosteroids (ICS)** in chronic obstructive pulmonary disease (COPD) (without an asthmatic component, eosinophils, <100 cells/μl, or pneumonia under ICS), such as:   - Budesonide - Fluticasone | Respiratory |
| **Montelukast** | Respiratory |
| **Oral corticosteroids** when used long-term in COPD patients, such as:   - Hydrocortisone - Prednisone | Respiratory |
| **Oral decongestants**, such as:   - Phenylephrine - Pseudoephedrine | Respiratory |
| **Roflumilast** | Respiratory |
| **Theophylline** | Respiratory |
| **Opioids** when used long-term in patients with chronic non-cancer pain, such as:   - Codeine - Fentanyl (transdermal patch) - Hydromorphone - Morphine - Oxycodone | Analgesics and co-analgesics |
| **Tapentadol** | Analgesics and co-analgesics |
| **Tramadol** | Analgesics and co-analgesics |
| **Meperidine/Pethidine** | Analgesics and co-analgesics |
| Long-term use of **NSAIDs** (>8 weeks) in patients with certain risk factors (e.g., history of GI ulcer, concurrent corticosteroid or anticoagulant use, cardiovascular disease, chronic kidney disease, heart failure, or hypertension), such as:  - Ibuprofen - Naproxen | Analgesics and co-analgesics |
| The following **NSAIDs** with less favorable risk-benefit profile:  - Acetylsalicylic acid (as analgesic) - Dexketoprofen - Diclofenac - Indomethacin - Ketoprofen - Ketorolac - Mefenamic acid - Meloxicam - Piroxicam - Tenoxicam | Analgesics and co-analgesics |
| **COX-2 inhibitors (coxibs)**, such as:  - Celecoxib - Etoricoxib - Parecoxib | Analgesics and co-analgesics |
| **Skeletal muscle relaxants** used for musculoskeletal pain, such as:  - Baclofen (excluding use for spasticity) - Cyclobenzaprine - Mephenesin - Methocarbamol - Tizanidine (excluding use for spasticity) - Thiocolchicoside | Musculoskeletal |
| **Capsaicin** (topical formulations) | Musculoskeletal |
| **Glucosamine** | Musculoskeletal |
