# Supplementary material for: Identification of Potentially Inappropriate Medications for Adults Below 65 Years: Protocol for a Modified Delphi Study
Source: JMIR Res Protoc. 2026 Jul 3;15:e92082. doi: 10.2196/92082 (PMC13331326; doi:10.2196/92082)
Supplement: Multimedia Appendix 2 [file resprot-v15-e92082-s002.docx]

**MULTIMEDIA APPENDIX 2: SAMPLE ROUND ONE QUESTIONNAIRE**

**A Modified Delphi Study to Identify Potentially Inappropriate Medications for Adults Under 65**

Thank you for agreeing to participate in this important initiative. The primary objective of this study is to develop a consensus-based list of potentially inappropriate medications (PIMs) for adults aged 18–65 through a modified Delphi process. This list will inform the creation of evidence-based clinical guidelines for the identified medications and medication classes. By focusing on medications with the highest potential for harm, the study seeks to ensure that younger patients benefit from proactive deprescribing efforts, complementing existing recommendations for older adults and addressing a critical gap in the landscape of polypharmacy management.

As a reminder, the Delphi process is a structured, iterative method used to gather expert insights and build consensus on complex issues. For this project, it will help identify and prioritize potentially inappropriate medications or medication classes for the adult population, ensuring they are informed by the collective expertise of a diverse panel.

Each survey round will include clear instructions, criteria to guide your responses, and targeted questions for input. Your responses will remain confidential and only aggregated data will be shared with the panel. No individuals will be identified when sharing the findings to allow you to freely express your opinions. Your participation in each round is essential to achieving meaningful and well-informed consensus. Completing all rounds ensures that the final priorities truly reflect the collective judgment and expertise of the panel, guiding the development of impactful clinical guidelines.

**Round One: Initial Prioritization and Input**

**Section 1: Instructions**

This modified Delphi study aims to identify medications that may be potentially inappropriate due to their risk-benefit profile. However, it does not aim to create a comprehensive list of all possible PIMs. Instead, we are focusing on medications that are:

- Commonly prescribed in adults under 65.
- Frequently associated with problematic prescribing patterns, safety concerns, or guideline inconsistencies.
- Relevant across broad medical conditions rather than highly specific to a single disease.

**Scope and Exclusions**

To maintain a focused and clinically relevant approach, this process does not include:

- **Medications used exclusively in pregnancy, pediatrics, or rare conditions**, as the goal is to address widespread prescribing concerns in adults.
- **Drug-drug interactions** that are purely pharmacokinetic/metabolic (e.g., CYP450 interactions), unless they represent a major prescribing issue.
- **Condition-specific recommendations**. While certain medications are considered inappropriate in specific clinical scenarios, this process will prioritize identifying medications with broader concerns across populations. Disease-specific considerations may be addressed in later phases of guideline development.
- Medication combinations that require monitoring but are not inherently inappropriate.
- Treatment duration, dosing adjustments, and individual patient factors. These will be considered in later stages of guideline development.

In this first round, you will assess a list of medications or medication classes based on their **potential benefit for inclusion in guideline development**. Your feedback will help us focus on areas where guidance is most needed.

Consider the following criteria and questions when rating each medication or class:

1. **Relevance to guideline inclusion**: Does inclusion of this medication in a clinical guideline address a critical issue, leading to meaningful improvements in patient outcomes?
2. **Balance of benefits and harms**: Does the harm of continuing this medication (e.g., adverse effects, pill burden) outweigh its benefits, particularly for adults?
3. **Relevance to patient preferences and values**: How likely is addressing the appropriateness of this medication to align with patients' goals, priorities, and quality-of-life considerations?
4. **Availability of alternatives**: Are there suitable alternatives (e.g., non-pharmacological options or safer medications) that could replace this medication if it were stopped?

For each medication or class, you will:

1. **Rate relevance**: Use a scale from **1** (Not relevant) to **5** (Extremely relevant) to indicate how relevant a medication or medication class is to be considered as a PIM for adults aged 18–65 given different scenarios.
2. **Provide justifications**: Use the open-text box to share any additional comments or considerations that may clarify your ratings.
3. **Provide suggestions**: Use the open-text box to suggest any additional medications or medication classes you believe should be included.

We encourage you to provide a response for each medication or medication class presented in the questionnaire. If you feel that you have limited knowledge about a specific medication or medication class and cannot confidently provide a rating, you may select the option "Insufficient knowledge to evaluate." However, we strongly encourage you to provide a rating, as every response contributes to the overall rigor and depth of the consensus process.

After completing this round, we will summarize the group’s responses and share these results with you. In subsequent rounds, you’ll have an opportunity to revise your priorities in light of group feedback.

Your input is invaluable in guiding the development of evidence-based, practical clinical guidelines. **The closing date for responses is [DAY-MONTH-YEAR].**

You can contact the research team at [**[EMAIL]**](mailto:eva.filosa@mail.mcgill.ca) if you need further information. Thank you for your time and commitment to this important work!

**Section 2: Demographics**

*First Name:* ____________________________________

*Last Name:* ____________________________________

*Email Address:* __________________________________

*Primary role(s) (please check all that apply):*

- Family physician
- Subspecialty physician
- Nurse or Nurse practitioner
- Pharmacist
- Researcher
- Guideline expert
- Person with lived experience, patient partner, or member of the public
- Other: ___________________________

*Years of experience in your respective field:*

- Less than 5
- 5-10
- 11-15
- 16-20
- 21-25
- More than 25
- Not applicable

*Which of the following best describes how you identify yourself:*

- Man
- Woman
- Non-binary
- Other: ___________________________

*Age:*

- Under 29
- 30-39
- 40-49
- 50-59
- 60-69
- 70 and older

*Ethnic background (please check all that apply):*

- Asian - Eastern
- Asian - Southeastern
- Black or African American
- Hispanic or Latino
- Native American or Indigenous
- Native Hawaiian or Other Pacific Islander
- White/Caucasian
- Other: ___________________________
- Prefer not to answer

*Country/Region of residence (please feel free to specify a city, region, province, territory or First Nation):*

________________________________________

**Section 3: Relevance as a PIM**

For each medication or medication class, please indicate how relevant it is to be considered as a PIM for adults aged 18–65 in the following scenarios. For certain medications or medication classes, simply indicate how relevant it is to be considered as a PIM for adults aged 18–65 given the context.

There is an open-ended comment box for each medication or medication class if you have optional comments you’d like to share with us.

**Guidance on Rating Scale and Approach**

As you complete this questionnaire, please use the 1–5 Likert scale to assess the relevance of each medication or medication class as a potentially inappropriate medication (PIM) for adults aged 18–65.

- **1** = Not relevant → This means the medication should not be considered at all as a PIM.
- **5** = Extremely relevant → This indicates the medication is highly relevant and should be strongly considered as a PIM.

We encourage you to trust your initial instinct when selecting a rating, as your expertise and first impression are valuable in refining the consensus. There is no need to overthink your responses, go with what feels most appropriate based on your knowledge and experience.

Medication A


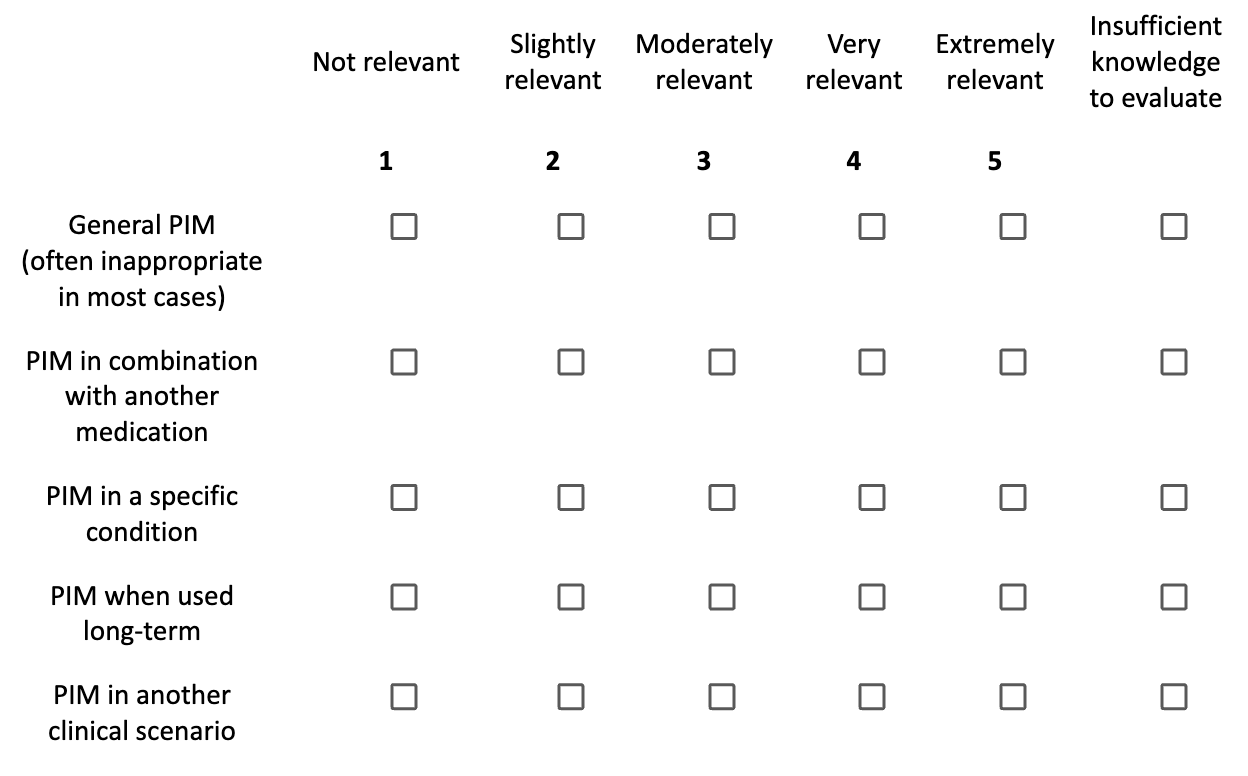


Medication B


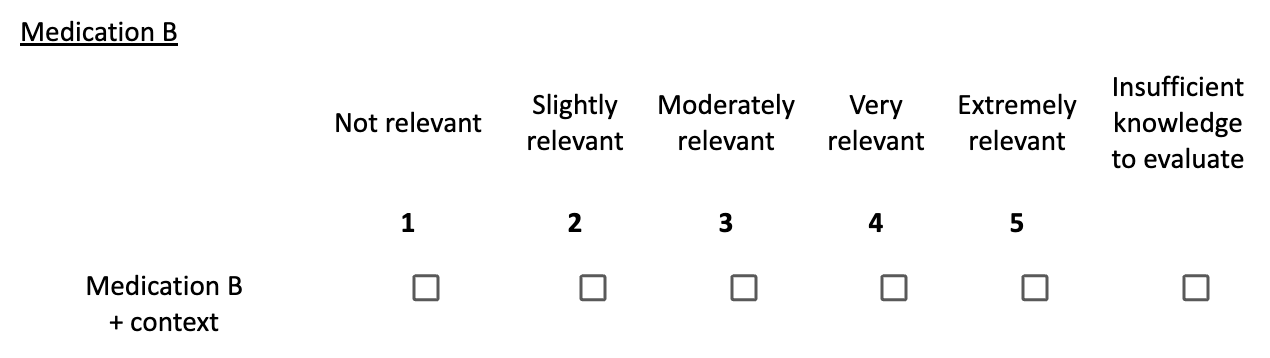


Medication C

[...]

**Section 4: Free-text comments**

Are there any additional medications or medication classes you believe should be included? Please specify and explain why.

|  |
| --- |

**MULTIMEDIA APPENDIX 2: SAMPLE ROUND TWO QUESTIONNAIRE**

**A Modified Delphi Study to Identify Potentially Inappropriate Medications for Adults Under 65 – Round Two: Reassessing PIMs**

**Section 1: Instructions**

Thank you for your continued participation! Based on the feedback from round one, we have summarized the group’s ratings and included additional medications suggested by the panel. This round focuses on refining our consensus and considering additional context-dependent concerns.

**Key updates based on round one**

- Medications were included if they showed high relevance scores with no disagreement (i.e., consensus for inclusion) in round one.
- Medications were excluded if they showed low relevance scores with no disagreement (i.e., consensus for exclusion) in round one.
- Certain newly suggested medications from round one will be rated for the first time in round two.
- Medications with no consensus (i.e., disagreement) remain for re-rating.

**Your role in this round is to:**

1. **Review results**

You will see the overall round one results for each medication or medication class. Also, new medications and medication classes may have been added based on suggestions from round one. Use this information to guide your rankings.

1. **Rate relevance**

From the revised list of medications with/without context specifications, please use a scale from **1** (Not relevant) to **5** (Extremely relevant) to indicate how relevant it is to be considered as a PIM for adults aged 18–65 given different scenarios.

1. **Provide justifications**

There is an open-ended comment box for each medication or medication class to justify any changes you might have made from the previous and to add any comments you’d like to share with us.

We will summarize the group’s responses and share these results with you in the subsequent round. **The closing date for responses is [DAY-MONTH-YEAR].**

You can contact the research team at **[EMAIL]** if you need further information.

Thank you for participating!

**Section 2: Round one results**

*Display of round one results.*

**Section 3: Relevance as a PIM**

For each medication or medication class, please indicate how relevant it is to be considered as a PIM for adults aged 18–65 in the following scenarios. For certain medications or medication classes, simply indicate how relevant it is to be considered as a PIM for adults aged 18–65 given the context.

There is an open-ended comment box for each medication or medication class if you have optional comments you’d like to share with us.

**Guidance on Rating Scale and Approach**

As you complete this questionnaire, please use the 1–5 Likert scale to assess the relevance of each medication or medication class as a potentially inappropriate medication (PIM) for adults aged 18–65.

- **1** = Not relevant → This means the medication should not be considered at all as a PIM.
- **5** = Extremely relevant → This indicates the medication is highly relevant and should be strongly considered as a PIM.

We encourage you to trust your initial instinct when selecting a rating, as your expertise and first impression are valuable in refining the consensus. There is no need to overthink your responses, go with what feels most appropriate based on your knowledge and experience.

Medication A


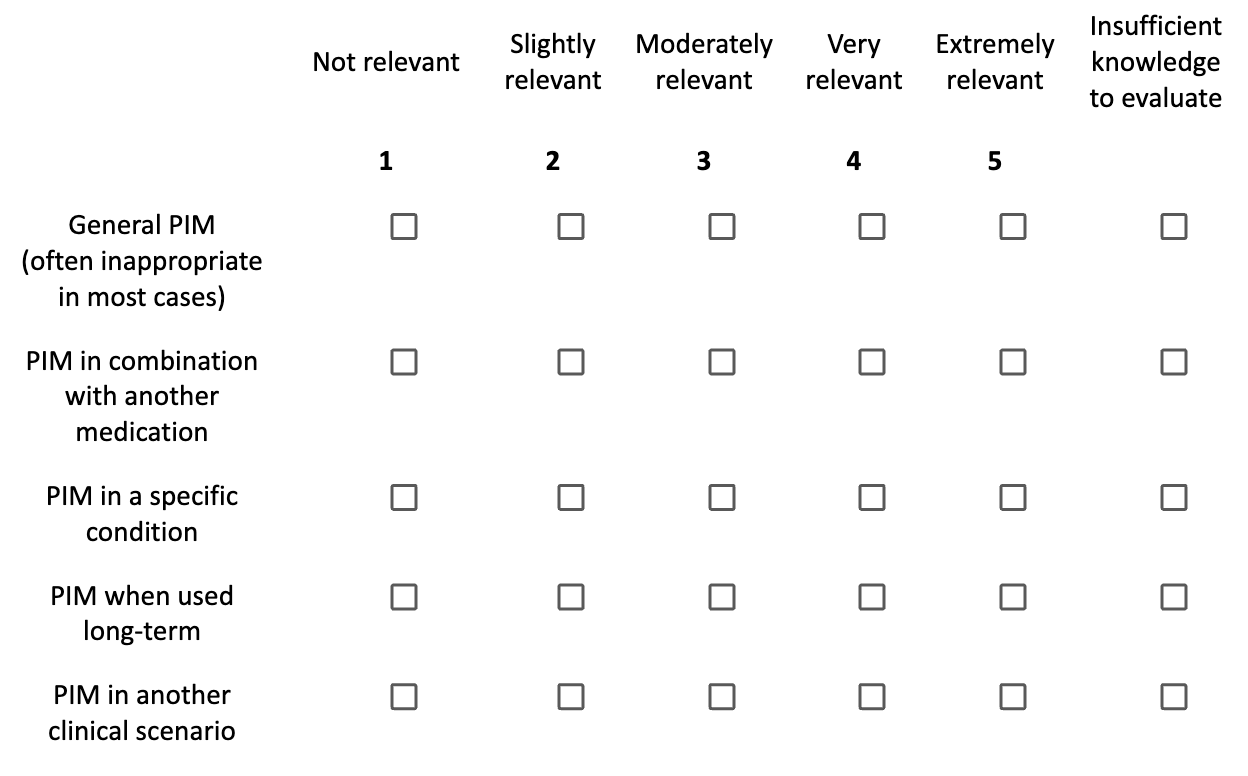


Medication B


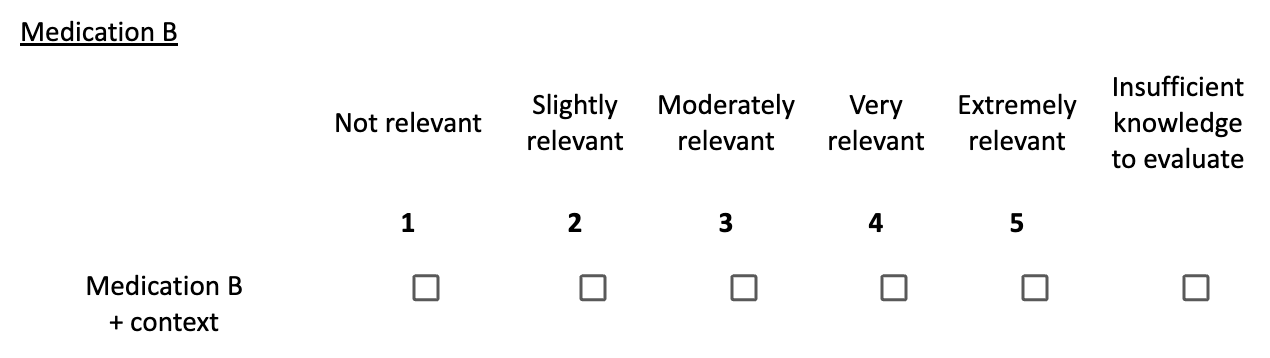


Medication C

[...]

**Section 4: Free-text comments**

Are there any additional comments you would like to share with us?

|  |
| --- |

**MULTIMEDIA APPENDIX 2: SAMPLE ROUND THREE QUESTIONNAIRE**

**A Modified Delphi Study to Identify Potentially Inappropriate Medications for Adults Under 65 – Round Three: Final Refinement**

**Section 1: Instructions**

Thank you for your continued participation! Based on the feedback from round two, we have refined the list of PIMs for adults aged 18–65. This round focuses on achieving final consensus on medications and their relevant contexts where disagreement persisted in round two.

**Key updates based on round two:**

- Medications were included if they showed high relevance scores with no disagreement (i.e., consensus for inclusion) in round two.
- Medications were excluded if they showed low relevance scores with no disagreement (i.e., consensus for exclusion) in round two.
- Medications with no consensus (i.e., disagreement) remain for re-rating.

**Your role in this round is to:**

1. **Review results**

You will see the overall round two results for each medication or medication class. Use this information to guide your final rankings.

1. **Rate relevance**

From the revised list of medications with/without context specifications, please use a scale from **1** (Not relevant) to **5** (Extremely relevant) to indicate how relevant it is to be considered as a PIM for adults aged 18–65 given different scenarios.

1. **Provide justifications**

There is an open-ended comment box for each medication or medication class to justify any changes you might have made from the previous and to add any comments you’d like to share with us.

Thank you for completing the final round of this Delphi process. **The closing date for responses is [DAY-MONTH-YEAR].** The results from this round will guide the development of an evidence-based clinical guideline aimed at improving patient care and clinical decision-making. We deeply appreciate your time and commitment to this important initiative. A summary of the findings and next steps will be shared with participants in the coming weeks.

You can contact the research team at **[EMAIL]** if you need further information. Thank you for your time and commitment to this important work!

**Section 2: Round Two results**

*Display of round two results.*

**Section 3: Relevance as a PIM**

For each medication or medication class, please indicate how relevant it is to be considered as a PIM for adults aged 18–65 in the following scenarios. For certain medications or medication classes, simply indicate how relevant it is to be considered as a PIM for adults aged 18–65 given the context.

There is an open-ended comment box for each medication or medication class if you have optional comments you’d like to share with us.

**Guidance on Rating Scale and Approach**

As you complete this questionnaire, please use the 1–5 Likert scale to assess the relevance of each medication or medication class as a potentially inappropriate medication (PIM) for adults aged 18–65.

- **1** = Not relevant → This means the medication should not be considered at all as a PIM.
- **5** = Extremely relevant → This indicates the medication is highly relevant and should be strongly considered as a PIM.

We encourage you to trust your initial instinct when selecting a rating, as your expertise and first impression are valuable in refining the consensus. There is no need to overthink your responses, go with what feels most appropriate based on your knowledge and experience.

Medication A


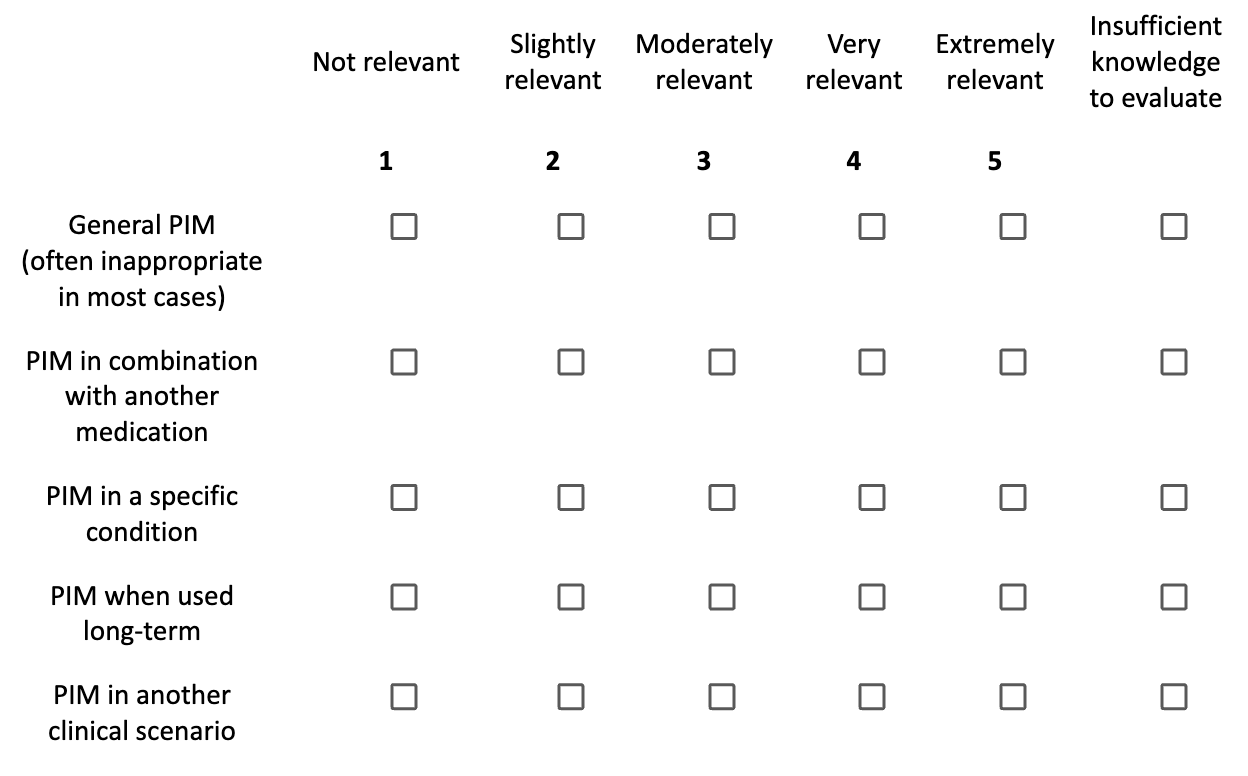


Medication B


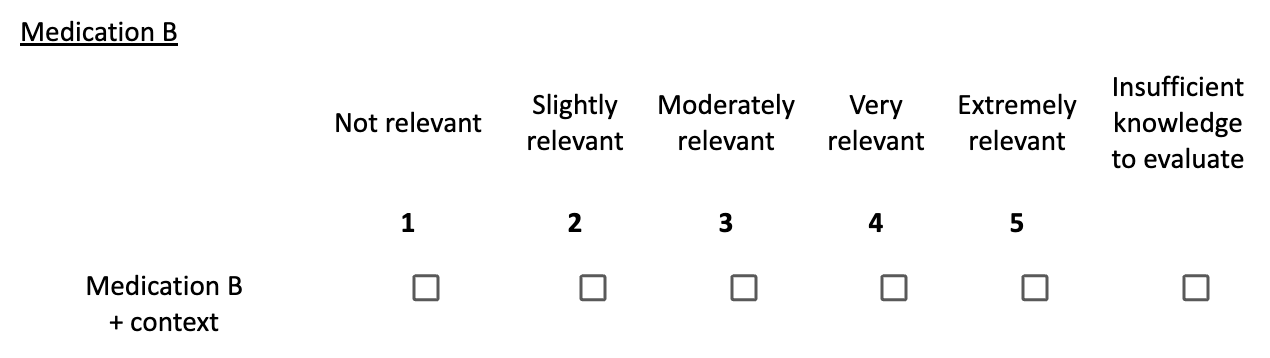


Medication C

[...]

**Section 4: Free-text comments**

Are there any additional comments you would like to share with us?

|  |
| --- |
